# Elucidation of the Effects of Heat Treatment on Polyphenolic Compounds in Highland Barley and Their Potential Mechanisms of Action in Improving Hypertension Using Targeted Metabolomics, Network Pharmacology, and Molecular Docking

**DOI:** 10.3390/foods15122095

**Published:** 2026-06-10

**Authors:** Zhengtao Wu, Yong Guan, Yanan Pan, Jianwen Zhang, Zhendong Liu, Erhao Zhang, Liang Li, Yuwei Yuan

**Affiliations:** 1 Key Laboratory of Deep Processing and High-Value Utilization of Characteristic Agricultural and Livestock Products of Xizang Autonomous Region, Food Science College, Xizang Agriculture & Animal Husbandry University, Nyingchi 860000, China; 2 State Key Laboratory for Quality and Safety of Agro-Products, Zhejiang Academy of Agricultural Sciences, Hangzhou 310021, China （Y.Y.); 3 Key Laboratory of Information Traceability for Agricultural Products, Ministry of Agriculture and Rural Affairs of China, Institute of Agro-Products Safety and Nutrition, Zhejiang Academy of Agricultural Sciences, Hangzhou 310021, China

**Keywords:** highland barley, network pharmacology, hypertension, antioxidant activity, thermal processing

## Abstract

This study aims to systematically elucidate the influence of various heat treatment methods on the phenolic compounds in highland barley and their potential antihypertensive processes via chemical, in vitro bioactivity, and bioinformatics prediction analyses. This work employed UHPLC-Q Exactive HFX-MS/MS targeted metabolomics technology to ascertain metabolites in barley treated with five different thermal conditions: steaming (ST), boiling at atmospheric pressure (BO), boiling at high pressure (PO), extrusion puffing (EX), and sand-roasting (SR). The data revealed 252 phenolic metabolites, comprising 19 phenolic acids and 233 flavonoids. Moreover, it was observed that, in comparison to the untreated group, various heat treatments yielded substantial differences in the profiles of phenolic compounds. Notably, extrusion puffing (EX) exhibited superior performance: it increased specific flavonoid glycosides such as Clitorin and Quercetin 3-O-rutinoside-(1-2)-O-rhamnoside, while also improving direct antioxidant capabilities such as DPPH and FRAP. In addition, network pharmacology analysis of differentially expressed metabolites in the puffed group identified 44 potential targets, including TNF, IL-6, MMP-9, HIF-1A, and ACE. The KEGG and GO enrichment analyses revealed a substantial enrichment of these targets in classic hypertension-related pathways, including lipid metabolism, atherosclerosis and fluid shear stress. The molecular docking findings indicated that Apigenin 7-O-(2G-rhamnosyl) gentiobioside had significant binding affinities for the target proteins MMP9 and ACE. This study demonstrated that EX is an efficient processing method, with highland barley polyphenols showing potential antihypertensive activity. The findings provide a novel theoretical foundation and research direction for optimizing highland barley processing to maximize functional component utilization and elucidate its food-derived antihypertensive mechanisms.

## 1. Introduction

Highland barley (*Hordeum vulgare* L. var. *nudum* Hook. f) is the primary food for the population in the Qinghai–Tibet Plateau region. Its abundance of bioactive compounds, including polyphenols, dietary fiber and β-glucan, leads to beneficial effects, including the regulation of blood pressure and blood lipid and glucose levels [1,2,3], thus making it an essential raw material in functional food formulations. Bioactive polyphenols are widely found in grains, vegetables, and fruit [4], with antioxidant [5], anti-inflammatory [6], and blood glucose/lipid-regulating effects [7]. Because of its unique chemical composition and biological properties, polyphenol-rich highland barley is an essential source of phenolic acids and flavonoids for human consumption. However, its rough texture and restricted palatability make it challenging for most individuals to implement it in their daily lives. At present, only 2% of highland barley is utilized to produce highland barley-based meals [8]. The literature suggests that processing enhances the digestibility and absorption of barley, increasing its edible value, nutritional content, and functional components [9]. Moreover, different processing methods exert distinct effects on the quality and composition of highland barley. Wang et al. [10] discovered that thermal processing, specifically boiling and baking, influences the polyphenolic compounds in highland barley, where these processes act as non-chemical approaches to enhance phenolic content in whole grains. Yang et al. [11] compared thermal processing methods (cooking, puffing, roasting, fermentation) with non-thermal procedures, revealing that these approaches substantially modify the nutritional value, bioactive components, and polyphenol content of highland barley. Accordingly, this study treated highland barley with five thermal processing methods: steaming (ST), atmospheric-pressure boiling (BO), high-pressure boiling (PO), sand-roasting (SR), and extrusion puffing (EX), to explore changes in its bioactive components and antioxidant capacity.

Hypertension remains the primary risk factor for cardiovascular disorders and the highest risk factor for global mortality. It affects 1.28 billion individuals worldwide and causes annual healthcare expenditures of over $370 billion [12]. Globally, both the elderly and children are at risk of developing hypertension, with the affected population gradually increasing [13]. The current clinical management of hypertension predominantly depends on medication; however, antihypertensive drugs could place a financial strain on ordinary households in low- and middle-income nations [14], and their prolonged use may be correlated with adverse effects and patient dependency concerns. Given the important role of diet in blood pressure homeostasis, Dai et al. [15] recommend dietary methods as a practical and acceptable strategy for managing blood pressure. Benisi-Kohansal et al. [16] assert that whole grains significantly mitigate the risk of numerous diseases, including cardiovascular disease, obesity, and cancer. Dietary interventions based on staple foods not only correspond more closely with daily routines and are easier to sustain over time, but also facilitate the early prevention and management of chronic diseases while ensuring sufficient nutritional intake. This method corresponds with the health philosophy that food and medication originate from the same source, offering a secure, cost-effective, and sustainable complementary strategy for preventing and treating chronic metabolic disorders such as hypertension. Recent studies indicate that polyphenols have potent antioxidant capabilities [17,18,19]. Oxidative stress, a principal contributor to hypertension, can affect the renin–angiotensin system, the endothelial system, and genetic polymorphism, thereby raising the likelihood of hypertension. Fraga et al. [20] observed that polyphenolic chemicals contribute positively to the management of coronary atherosclerotic heart disease (CAD), hypertension, diabetes, and various other disorders. Therefore, this study hypothesizes that polyphenols from highland barley may alleviate hypertension.

Network pharmacology is an analytical approach for investigating the interactions within the “drug–disease–target” model [21], and enables the characterization of drug properties. Specifically, it links disease features, bioactive components or pharmacological targets with metabolites or signaling pathways [22]. Furthermore, it evaluates relationships among molecules, and is thus invaluable for drug discovery and assessing structure-activity associations [23]. These methods enable the efficient and convenient identification of specific functional components from complex mixtures. For example, Younis et al. [24] revealed the metabolomic variations in quinoa grains from different geographical origins and the material basis of their anti-Alzheimer’s disease activity using GC-MS, LC-QTOF-MS/MS, molecular networking, and chemometric analysis. Hang et al. [25] uncovered the potential mechanism of celery seeds in the treatment of gout via network pharmacology, molecular docking, and molecular dynamics simulation, and clarified their key bioactive components and core signaling pathways. Therefore, the current work applied network pharmacology as well as molecular docking to evaluate the mechanisms responsible for the actions of highland barley polyphenols in hypertension.

This research employed UHPLC-Q Exactive HFX/MS to investigate alterations in polyphenolic compounds in highland barley treated with five distinct processing methods: ST, BO, PO, EX, and SR. In addition, the influence of heat treatment on the antioxidant activity of highland barley was also evaluated to ascertain which processing method most effectively maintains the bioactive constituents of highland barley. Moreover, this research utilized molecular docking and network pharmacology to evaluate the mechanisms by which significant antioxidant phenolic compounds in highland barley polyphenols may alleviate hypertension, offering a theoretical foundation for predicting the mechanisms through which highland barley polyphenols improve hypertension management.

## 2. Materials and Methods

### 2.1. Samples and Chemicals

Highland barley (Zangqing 16). Analytical grade ethanol, hydrochloric acid, ethyl acetate, n-hexane, and Folin–Ciocalteu reagent were acquired from Yuanye Biotechnology Co., Ltd. (Shanghai, China). 1,10-Phenanthroline was provided by Shanghai Shanpu Chemical Co., Ltd. (Shanghai, China). Methanol (chromatographic grade) was supplied by Thermo Fisher Scientific (USA). Total phenol (TP) and total flavonoid (TF) kits, as well as kits for evaluating overall antioxidant (via DPPH, FRAP, and ABTS methods) and hydroxyl radical (•OH) scavenging capacities, were acquired from Suzhou Keming Biotechnology Co., Ltd. (Suzhou, China).

### 2.2. Highland Barley Processing

The different methods were pretested to ascertain the conditions of ST, EX, SR, BO, and PO. The barley was thoroughly cooked in all cases. The specific treatments are as follows: (1) Raw (CK): The impurities in highland barley grains were removed, and defective or empty grains were excluded. The grains were subsequently washed and air-dried. (2) EX: Untreated highland barley was milled using an 800 A grinder (Dongguan Huatai Electric Appliance Co., Ltd., Dongguan, China) and thereafter passed via a 60-mesh screen. Subsequently, the sample (3 kg) was uniformly combined with water to attain a moisture content of 6%. The extrusion was conducted with an extruder (DSE-65-I, Jinan Dingrun Machinery Equipment Co., Ltd., Jinan, China), with the 100 °C and 140 °C temperatures of the middle and rear sections, respectively. The rotary cutting frequency was 27 Hz, and the feeding rate was 14 kg/h, which yielded extruded puffed highland barley. (3) SR: Highland barley grains were hydrated with water for 10 min, drained, and subsequently placed in a preheated stir-frying apparatus (J-03, Hefei Yijiu Electromechanical Technology Co., Ltd., Hefei, China) at 260 °C. The grains were constantly stirred for 10 s until the majority expanded and burst. (4) ST: Highland barley was wrapped in gauze and steamed for 65 min at 120 °C (EZ28BS10, Zhejiang Supor Co., Ltd., Yuhuan, China). (5) BO: 4000 mL of water was heated with an induction cooker (C21-WT2112T, Guangdong Midea Life Electric Appliance Manufacturing Co., Ltd., Foshan, China) at 2100 W. Once the water reached boiling point, highland barley grains were added for 55 min until most grains were fully cooked. (6) PO: water (4000 mL) was transferred into a pressure cooker (A26-9.5-80, Zhuhai Shuangxi Electric Appliance Co., Ltd., Zhuhai, China) and heated using an induction cooker at 2100 W. Then, highland barley grains were added to the boiling water and cooked under high pressure for 25 min until most of the grains were thoroughly cooked. All samples, including the untreated control, were dried to a consistent weight in an electric thermostatic drying oven (TGF-9140A, Shanghai Zhetu Scientific Instrument Co., Ltd., Shanghai, China) at 30 °C, consistently milled with a grinder, filtered through a 100-mesh filter to yield whole highland barley flour, and preserved in a sealed desiccator in the dark for subsequent utilization.

### 2.3. Chemical Antioxidant Assays

Sample preparation for antioxidant activity assay: Approximately 0.1 g of highland barley sample was weighed, mixed with 1 mL of extraction solution, and homogenized under ice-bath conditions. The mixture was centrifuged at 10,000× *g* and 4 °C for 10 min, and the resulting supernatant was collected for subsequent determination.

Extraction of phenolic compound: Approximately 0.02 g of highland barley sample was weighed, added with 2 mL of extraction solution, and subjected to shaking extraction at 60 °C for 2 h. Following extraction, centrifugation was performed at 10,000× *g* and 25 °C for 10 min, and the supernatant was retained for testing.

Commercial assay kits were used to determine total phenolic, total flavonoid, as well as DPPH, FRAP, ABTS and •OH scavenging capacity. All results were calculated on a dry weight basis.

### 2.4. Metabolomics of Polyphenolic Metabolites in Highland Barley

#### 2.4.1. Metabolomics of Phenolic Acid Metabolites

The determination of phenolic acid metabolites was performed according to previously reported methods, with appropriate modifications [26,27,28]. Preparation of Standards: All standard compounds required for this experiment were accurately weighed and individually dissolved in methanol to prepare 10 mg/mL single standard stock solutions. Appropriate volumes of these stock solutions were mixed to prepare mixed standard working solutions at concentrations of 1 μg/mL or 10 μg/mL, which were stored for subsequent use. Sample Preparation: 100 mg of the sample was precisely weighed, added to 2 mL of 4 M sodium hydroxide solution for extraction, and hydrolyzed at 40 °C for 2 h. Then, the pH of the standard was kept at 2.0 using 4 M HCl. Subsequently, 2 mL of n-hexane was introduced at room temperature, shaken for 20 min, and then the n-hexane layer was discarded. Extraction of the aqueous layer was carried out with ethyl acetate (2 × 2 mL), and the mixture was vacuum-dried using a rotary evaporator (ZLS-1, Hunan Hexi Instrument Equipment Co., Ltd., Changsha, China) at 35 °C. Lastly, 0.2 mL of a 50% methanol–water solution was added and then analyzed.

Chromatography parameters: Column: Waters HSS T3 (50 × 2.1 mm, 1.8 μm); details of the gradient elution are given in Table 1. For mass spectrometry (MS), data were obtained with a high-resolution mass spectrometer (Q Exactive HFX, Thermo Fisher Scientific, Waltham, MA, USA), equipped with an electrospray ionization (ESI) source. The sheath gas flow was 40 arb, the auxiliary gas flow was 10 arb, the ion spray voltage was −2800 V, the temperature was 350 °C, and the ion transfer tube temperature was 320 °C. For negative ion detection, the scanning mode was Single Ion Monitoring (SIM). The m/z range for the initial scan was 80–350.

#### 2.4.2. Metabolomics of Flavonoid Metabolites

Preparation of Standards: All standard compounds required for this study were accurately weighed and individually dissolved in methanol to prepare 10 mmol/L single standard stock solutions. Appropriate volumes of these stock solutions were mixed to prepare a 10 μmol/L mixed standard working solution, which was stored for subsequent use. Sample preparation: Sample (100 mg) was transferred to the centrifuge tube, mixed with 500 μmol/L 70% methanol solution, vortexed, and then sonicated at room temperature (30 min). Then, the sample as centrifuged for 10 min (Eppendorf 5430R, Anhui Zhongke Zhongjia Scientific Instrument Co., Ltd., Hefei, China) at 12,000 rpm to collect the supernatant.

Chromatographic conditions: Column: Waters HSS T3 (100 × 2.1 mm, 1.8 μm); gradient elution parameters are provided in Table 2. For MS analysis, an ESI source was employed, sheath gas was 40 arb, auxiliary gas was 10 arb, ion spray voltage was −2800 V, temperature was 350 °C, and ion transfer tube temperature was 320 °C. The full scan-ddMS2 mode was utilized with positive ionization mode. First-stage mass scan range (scan m/z range) was 70–1050.

### 2.5. MS Data Analyses

The raw spectra acquired from the ultra-high-performance liquid chromatography–quadrupole/electrostatic field Orbitrap high-resolution MS system (UHPLC-Q Exactive HFX/MS, Thermo Fisher Scientific, Waltham, MA, USA) were preprocessed via TraceFinder software 5.1SP1 (ThermoFisher Scientific, Waltham, MA, USA). The preprocessing comprised baseline filtering, identification, and matching of peaks, leading to a data matrix including retention times, mass-to-charge ratios, and peak intensities. A total ion current chromatogram (TIC) graph was produced by continuously plotting the sum of all ion intensities in the spectra at each time point; the x-axis indicated retention times (min) and the y-axis showed ion current intensities (Intensity). The TraceFinder software was employed to generate a standard curve. Lastly, the compound’s absolute quantification was assessed via the external standard method.

### 2.6. Network Pharmacology

#### 2.6.1. Identification of Active Ingredient Targets

The SMILES numbers and SDF files of the differentially expressed metabolites determined via optimal processing methods in targeted metabolomics were acquired from PubChem (https://pubchem.ncbi.nlm.nih.gov/, accessed on 26 December 2025). Candidate targets of the metabolites were predicted using SEA (https://sea.bkslab.org/, accessed on 26 December 2025) and Swiss Target Prediction (http://www.swisstargetprediction.ch/, accessed on 27 December 2025). Targets predicted by both databases were consolidated, and duplicates were eliminated.

#### 2.6.2. Search for Hypertension Targets

The GeneCards (https://www.genecards.org/, score ≥ 10, accessed on 28 December 2025), OMIM (https://www.omim.org/, accessed on 28 December 2025), and TTD databases (https://db.idrblab.net/ttd/, accessed on 28 December 2025) were searched employing “Hypertension” as the keyword to identify targets linked to hypertension. All the identified targets were organized and compiled [29]. Subsequently, integration and deduplication were conducted to acquire the disease targets associated with the pathogenesis of hypertension.

#### 2.6.3. Identification of Targets Related to the Treatment of Hypertension by Active Components of Highland Barley Polyphenols

The active component targets were combined with disease targets via the Venny 2.1.0 (https://bioinfogp.cnb.csic.es/tools/venny/index.html, accessed on on on 13 January 2026) platform to determine their intersection, ultimately identifying the potential targets of highland barley polyphenols that mitigate hypertension. The Cytoscape 3.10.0 program was applied to produce a network of significant targets for hypertension improvement through the active components of highland barley polyphenols, with topological features analyzed utilizing tools such as “Network Analyzer”.

#### 2.6.4. Development of PPI Network and Identification of Key Targets

To further examine the mechanism by which active components of highland barley polyphenols mitigate hypertension, the hypertension improvement-related targets in polyphenols used to generate a protein–protein interaction (PPI) network in STRING (https://string-db.org/, Version 11.0, accessed 22 January 2026), using a minimum combined score of 0.400 and the species “*Homo sapiens*”. Targets lacking interactions were removed, and the data file containing PPI relationships was exported. Visualization of the network was performed using Cytoscape 3.10.0. Core targets among the intersection targets were identified by network topological analysis by integrating the following three indices: betweenness centrality, closeness centrality, and degree centrality.

#### 2.6.5. GO Function and KEGG Signaling Pathway Enrichment Analyses

GO enrichment of the identified targets for highland barley polyphenols’ active components for hypertension improvement was conducted utilizing the “Cluster Profiler” package in R. Gene annotations in the categories of biological process (BP), molecular function (MF), and cellular component (CC) were assessed using *p* < 0.05. KEGG pathway enrichment of the targets was performed using *p* < 0.05. The Microbioinformatics platform (https://www.bioinformatics.com.cn/, accessed 29 January 2026) was employed for the visualization analysis of primary biological functions and pathways.

### 2.7. Molecular Docking

The SDF files of metabolites were converted to Protein Data Bank (PDB, https://www.rcsb.org/, accessed on 5 February 2026) files using Open Babel 2.3.2. Protein structures were retrieved from the PDB. PyMOL 2.3.4 was utilized to conduct procedures, including dehydration and ligand removal, on the receptor proteins. The AutoDockTools software ADT1.5.6 was utilized to alter the receptor proteins (including hydrogenation and charge equilibration), and both the receptor proteins and small ligand molecules were changed to pdbqt format, respectively. Molecular docking of receptor proteins and small ligand molecules was conducted via AutoDock Vina 1.1.2, and the findings were displayed with PyMOL.

### 2.8. Statistical Analysis

Experiments were performed in triplicate, and data are presented as mean ± standard deviation and were compared using one-way ANOVA. The adjusted MS data were imported into SIMCA 14.1 software for OPLS-DA and principal component analysis (PCA). Data summary and initial processing were conducted via Microsoft Excel 2016, while one-way ANOVA was conducted in IBM SPSS Statiscs 27 .

## 3. Results and Discussion

### 3.1. Analysis of Flavonoid and Total Phenol Contents in Highland Barley with Different Treatments

**Figure 1:**
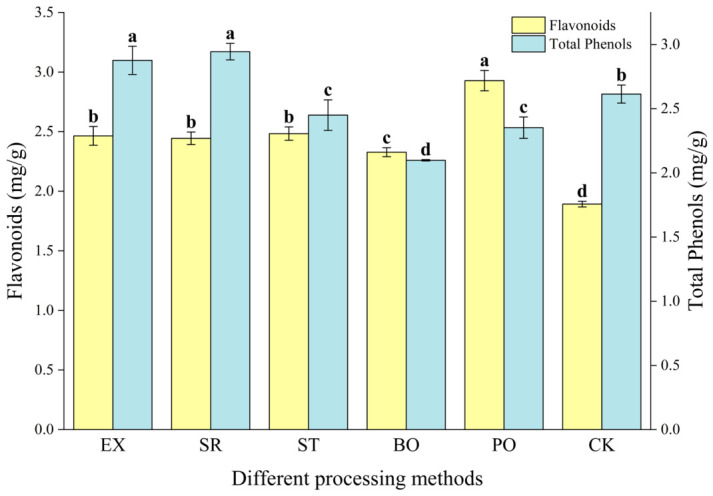
Contents of polyphenols and flavonoids in highland barley under different treatments. Note: EX: extrusion puffing, SR: sand-roasting, ST: steaming, BO: atmospheric-pressure boiling, PO: high-pressure boiling, CK: Raw. Different letters in the same column indicate significant differences (*p* < 0.05).

Figure 1 illustrates the flavonoid and total phenolic concentrations of highland barley processed using various techniques. The flavonoid concentration in all processed samples surpassed that of untreated highland barley; the highest content was recorded in the PO-treated highland barley at 2.93 ± 0.09 mg/g, followed by the ST-treated at 2.48 ± 0.06 mg/g, EX-treated at 2.46 ± 0.08 mg/g, SR-treated at 2.44 ± 0.05 mg/g, and BO-treated at 2.33 ± 0.04 mg/g. The minimum concentration was recorded in untreated highland barley at 1.89 ± 0.02 mg/g. An analysis indicates that the improvement of flavonoid content may be attributed to thermal treatment, which disrupts plant cell walls, improving the extraction efficiency of bound flavonoid compounds and releasing these bound flavonoids, thereby leading to increased flavonoid detection [30]. Furthermore, EX and SR treatments enhanced detectable polyphenol content, measuring 2.88 ± 0.11 and 2.94 ± 0.06 mg/g, respectively. ST, BO, and PO treatments indicated 2.45 ± 0.12, 2.1 ± 0.01, and 2.35 ± 0.08 mg/g values, all of which were less than the CK-treated highland barley (2.61 ± 0.07 mg/g). The underlying mechanism may involve the temperatures during EX- and SR- treatments reaching the threshold necessary for the release of bound phenols, which increases the detectable total phenolic concentration. ST, BO, and PO treatments were unable to reach the temperature necessary for the release of bound phenol, leading to the loss of phenolic compounds during processing and resulting in reduced concentrations compared to untreated highland barley. Paucar-Menacho et al. [31] reported that cooking and steaming decreased the overall phenolic content in cereals, consistent with the current study’s findings. Moreover, Kataria et al. [32] discovered that roasting and microwave treatment enhanced total phenolic and total flavonoid in teff, corroborating the presented findings.

### 3.2. Analysis of Antioxidant Capacity of Highland Barley Under Different Heat Treatments

**Figure 2:**
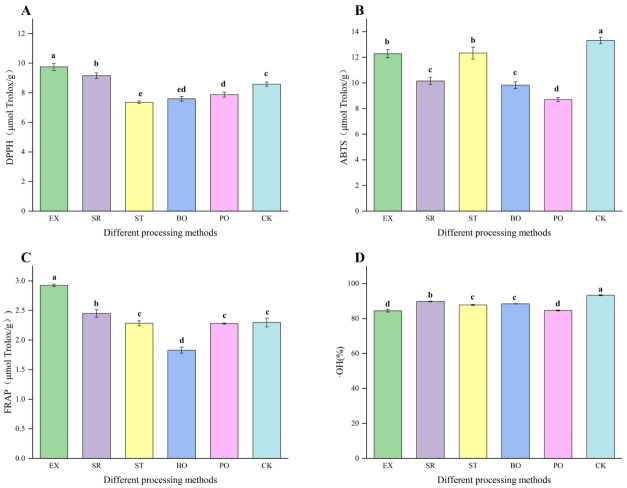
Antioxidant Capacity of Highland Barley under Different Processing Methods. Note: EX: extrusion puffing, SR: sand-roasting, ST: steaming, BO: atmospheric-pressure boiling, PO: high-pressure boiling, CK: Raw. Different letters in the same column indicate significant differences (*p* < 0.05).

The antioxidant abilities of the treated highland barley samples were assessed using four distinct methodologies: DPPH, FRAP, ABTS, and hydroxyl radical scavenging capacity assays. The in vitro antioxidant activities exhibited no consistent pattern, and the findings are provided in Figure 2 and Supplementary Table S1.

DPPH is a free radical scavenger frequently employed in assays to evaluate antioxidant activity. The FRAP method is a widely utilized assay for evaluating the reducing capacity of antioxidants in samples. Figure 2 illustrates substantial variations in antioxidant capacity among highland barley samples subjected to different heat treatments (*p* < 0.05). Zhou et al. [33] indicated that the concentration of total phenols influences DPPH radical scavenging activity. Švetková et al. [34] showed that the concentration of polyphenolic polymers affects FRAP antioxidant ability. The findings indicated that the DPPH radical scavenging rate and FRAP antioxidant capacity of EX and SR treatments were superior to those of untreated highland barley, whereas the antioxidant capabilities of ST, BO, and PO treatments were inferior to those of the CK group. Bai et al. [35] similarly observed an elevation in DPPH radical scavenging capacity of highland barley after heat treatment. This can be ascribed to the enhanced total phenolic following EX and SR, which increased antioxidant activity. The reduced total phenolic observed in ST, BO, and PO treatments induced decreased antioxidant capacity relative to the CK group.

ABTS is a widely utilized probe for assessing antioxidant activity and hydroxyl radical scavenging ability. Figure 2 illustrates that several treatments significantly influenced the antioxidant activity estimated by the ABTS assay. The findings indicated that untreated highland barley showed the greatest ABTS radical scavenging activity, measuring 13.32 ± 0.25 μmol Trolox/g. All thermal treatments showed inhibitory effects on the ABTS radical scavenging activity of highland barley, with the PO-treated sample presenting the lowest value at 8.71 ± 0.16 μmol Trolox/g. The above results are in good accordance with the conclusions reported previously by Yang et al. [11]. This may be attributed to the decomposition of key active components responsible for ABTS radical scavenging activity in highland barley during thermal treatment, which led to a decrease in ABTS antioxidant capacity.

The hydroxyl radical scavenging rate is an essential metric for assessing the capacity of samples to neutralize hydroxyl radicals (•OH). A higher value signifies enhanced hydroxyl radical scavenging capability. Figure 2 illustrates that various heat treatments substantially influenced the hydroxyl radical scavenging rate of highland barley. The untreated group demonstrated the maximum scavenging rate at 93.32% ± 0.28%, whereas the EX-treated group exhibited the lowest rate at 84.45% ± 0.72%. All thermal processing procedures decreased the hydroxyl radical scavenging capacity of highland barley. The primary reason may be that thermal treatment promotes starch gelatinization in highland barley, resulting in the formation of macromolecular gelatinized starch. The detection of hydroxyl radicals occurs in an aqueous phase, where gelatinized starch can create a colloid with water and adsorb free radicals or detection reagents, leading to a reduction in the detected signal. The decrease in •OH scavenging rate was mainly due to the degradation of active substances closely associated with hydroxyl radical scavenging activity during thermal treatment. Wu et al. [36] indicated that both the ABTS radical scavenging activity and the hydroxyl radical scavenging capacity of quinoa were reduced following thermal treatment, aligning with the findings of the current investigation.

### 3.3. Targeted Metabolomics Profiling Results

#### 3.3.1. Targeted Metabolomics of Components

**Figure 3:**
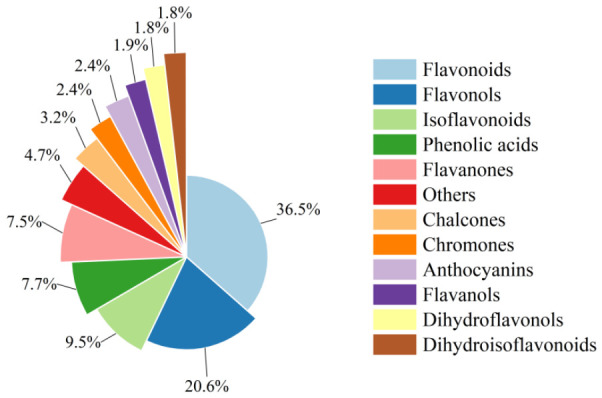
Proportions of substances detected by targeted metabolomics.

The concentrations of flavonoids and phenolic acids in highland barley subjected to various treatments were assessed by targeted metabolomics. Figure 3 and Supplementary Table S2 shows that 252 metabolites were identified in highland barley samples across all treatment groups, comprising 19 phenolic acid metabolites and 233 flavonoid metabolites. Figure 3 demonstrates the relative proportions of each category. Flavones comprised the largest share at 36.50%, followed by flavonols at 20.60%, isoflavones at 9.50%, and phenolic acids at 7.70%.

#### 3.3.2. Analysis of Metabolic Highland Barley Under Different Heat Treatments

**Figure 4:**
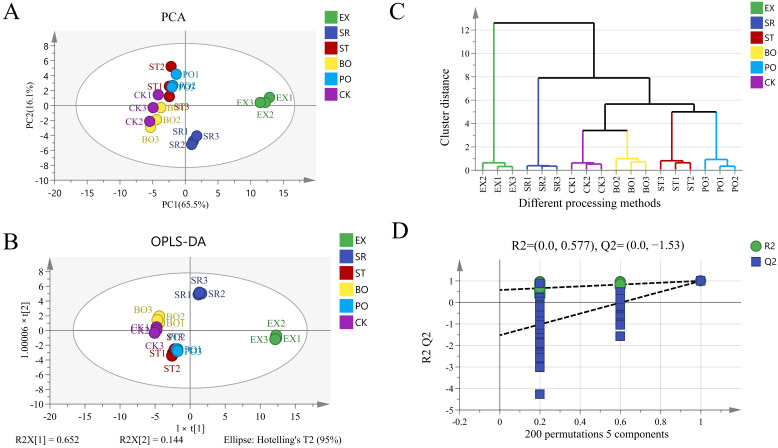
Multivariate statistical analysis of highland barley under different heat treatments. (**A**): PCA score plot; (**B**): HCA cluster analysis; (**C**): OPLS-DA score plot; (**D**): OPLS-DA permutation test. Note: EX: extrusion puffing, SR: sand-roasting, ST: steaming, BO: atmospheric-pressure boiling, PO: high-pressure boiling, CK: Raw.

PCA was conducted to decrease the dimensionality of the sample data collected under various dietary treatment circumstances, with each group consisting of three biological replicates to ensure data reliability. Figure 4A illustrates that principal component 1 (PC1) contributed to 65.5% of the variance, whereas principal component 2 (PC2) accounted for 21.6%, resulting in a total variance contribution rate of 87.1%. This signifies that these two main components represent the majority of the data information from the highland barley samples. Distinct clusters developed for several treatment groups, indicating considerable differences in metabolite composition among the groups and relatively moderate alterations within each group. This difference illustrates the significant influence of diverse heat treatments on the composition of highland barley samples. The EX group was distinctly separated from the other groups, demonstrating the highest level of divergence and underscoring its unique metabolic traits. The hierarchical clustering depicted in Figure 4B further confirms this finding, as the EX group was distinctly separated into an isolated cluster, highlighting its uniqueness in metabolite composition. To investigate the distinct differences among highland barley samples subjected to various treatments, this study developed a supervised OPLS-DA model to examine the similarities and disparities among the groups. Figure 4C,D indicate that the six groups of highland barley samples had a distinct separation trend in the OPLS-DA score plot, with a distribution pattern largely aligning with the PCA results, thus reinforcing the marked impact of varying heat treatments on the metabolite composition of highland barley. This study also performed 200 permutation tests to guarantee the model’s stability and dependability, mitigating overfitting. The test results indicated that the intercepts of R^2^ and Q^2^ were 0.577 and −1.53, respectively, signifying the lack of overfitting and showcasing the model’s robust prediction performance and stability.

#### 3.3.3. Analysis of Metabolite Characteristics in Highland Barley Under Different Treatment Methods

**Figure 5:**
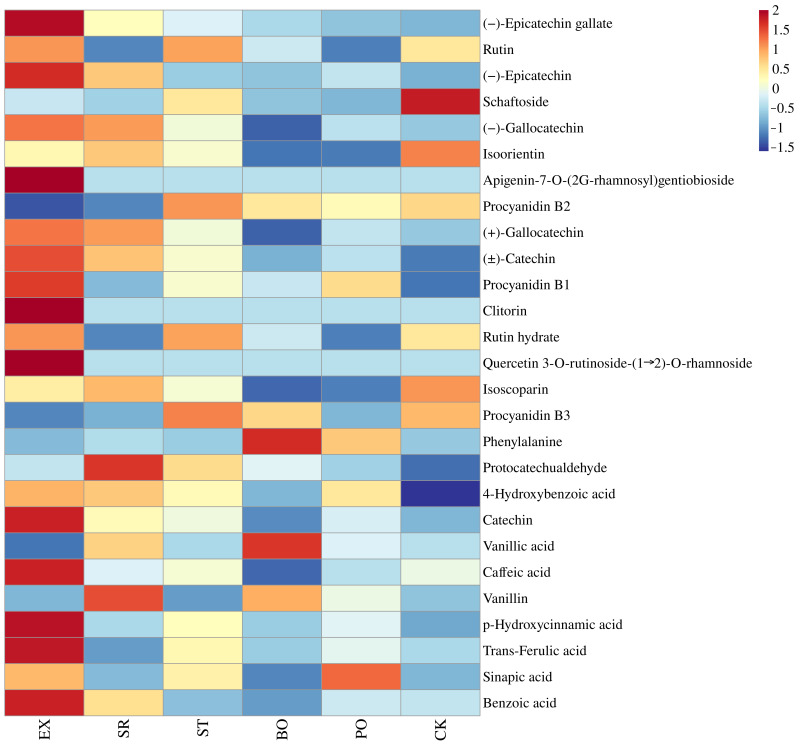
Clustered heatmap of metabolite contents under different heat treatments.

The variable importance in projection (VIP) values of metabolites in highland barley samples were determined. A total of 27 significant metabolites were identified using the criteria of VIP > 1 and *p* < 0.05 (Table 3). These included 9 phenolic acids, 11 flavonoids, 6 flavanols, and 1 amino acid. This suggests that various heat treatment approaches substantially affect metabolite production. Supplementary Figure S1 depicts the distribution of 27 major metabolites across various heat treatment procedures. The heatmap visualization analysis identified the relative abundance of metabolites. In the heatmap, dark squares signify high abundance, whereas light squares indicate low abundance, with color intensity reflecting the magnitude of variation. Significant differences are apparent among the highland barley groups treated to various treatments. Each of the six heat treatment procedures showed unique characteristics. Variations in time and temperature across these six approaches resulted in corresponding changes in certain metabolites. The majority of metabolites in the EX group display elevated expression levels, indicated by deep red hues (Figure 5). The concentrations of compounds like Clitorin, Apigenin-7-O-(2G-rhamnosyl) gentiobioside, Quercetin 3-O-rutinoside-(1-2)-O-rhamnoside, and (−)-Epicatechin gallate were markedly elevated in the EX group relative to other groups. This indicates that the expansion treatment facilitates the accumulation of a certain type of metabolites.

#### 3.3.4. Correlation Analysis

**Figure 6:**
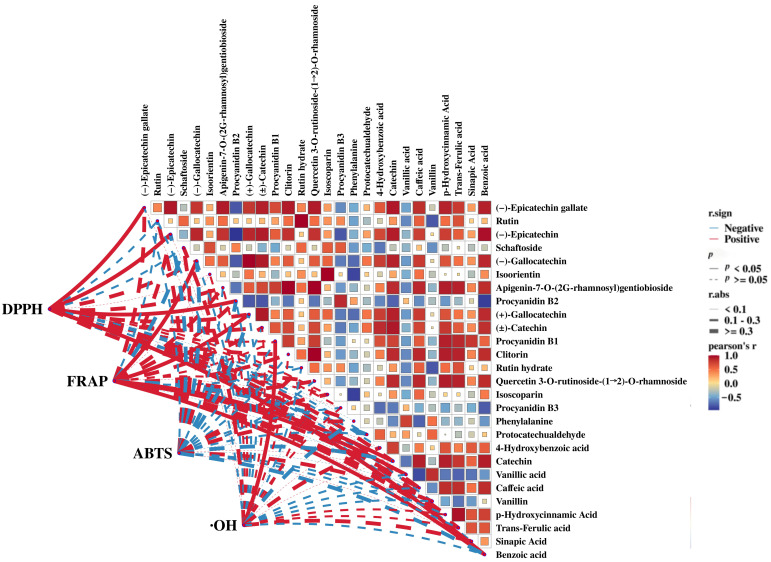
Correlation network heatmap of antioxidant capacity.

This study combined correlation network and heatmap analyses to more effectively evaluate the relationships between antioxidant capacity and significant metabolites. Figure 6 illustrates the relationships between essential metabolites and antioxidant ability from several perspectives. The majority of metabolites had a positive correlation with DPPH, FRAP, and ABTS antioxidant capabilities, while the hydroxyl radical scavenging capacity demonstrated a negative correlation with most metabolites. The correlation density between DPPH and FRAP was highest with the most intense coloration, as indicated in red in the heatmap. The connection densities of ABTS and hydroxyl radical scavenging capacity were comparatively decreased, with certain areas presenting a purple hue. (−)-Epicatechin gallate, (−)-Epicatechin and Procyanidin B2, together with benzoic acid, showed a significant positive correlation with DPPH values. (−)-Gallocatechin, (+)-Gallocatechin, catechin, vanillic acid and benzoic acid were significantly positively correlated with FRAP values. Procyanidin B1 had a significant positive correlation with •OH scavenging rate. Additionally, ABTS activity was positively correlated with rutin. The correlation trends between active substances and antioxidant capacities were not fully consistent, which was mainly attributed to the different action mechanisms of various antioxidant evaluation methods.

### 3.4. Network Pharmacological Analysis

#### 3.4.1. Prediction of Potential Targets of Highland Barley Polyphenols and Compound–Target Network Analysis

**Figure 7:**
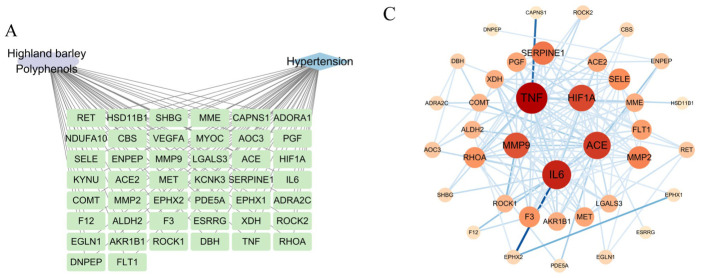

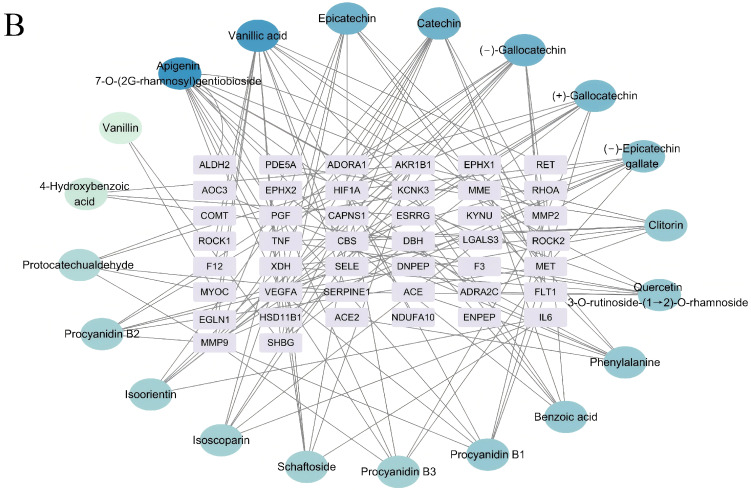
(**A**): Network diagram of highland barley polyphenol–disease–intersection targets; (**B**): Interaction network of highland barley polyphenols and intersection targets; (**C**): PPI network.

Network pharmacology was conducted to examine the antihypertensive activity of highland barley polyphenols for further mechanistic analysis. The aforementioned metabolomic data indicated that the extrusion processing group had the highest quantity of differential metabolites. Fu et al. [37] indicated that extrusion processing offers significant application potential in the production of high-value-added highland barley products. Therefore, 20 significantly divergent metabolites identified from the extrusion treatment were selected for further network pharmacological research (Supplementary Table S3). The SMILES and SDF files of each compound were successively obtained and uploaded to the corresponding target prediction databases. A total of 130 compound targets were identified from the Swiss Target Prediction database, and 503 compound targets were acquired from the SEA database. Following the removal of redundant targets, 549 non-redundant putative targets were identified for the discovered metabolites. The term “Hypertension” was employed to identify targets from the Gene Cards, TTD, and OMIM databases for hypertension-related target collection. In total, 267 hypertension-related genes with a Relevance score > 10 were identified from the Gene Cards database; 572 hypertension-related genes were extracted from the OMIM library; and 112 hypertension-related genes were sourced from the TTD database. After merging and deduplication removal, 888 unique therapeutic targets for hypertension were identified. The Venn diagram (Supplementary Figure S2) was generated using Venny 2.1.0 software. The diagram indicates 44 common targets, which were recognized as possible targets responsible for the antihypertensive effects of highland barley polyphenols. Figure 7A presents the comprehensive details of these overlapping targets.

In total, 44 common targets were recognized as the functional targets of highland barley polyphenols for ameliorating hypertension and were imported into Cytoscape 3.10.0 to generate a “compound–target” interaction network (Figure 7B). In the PPI network, an increased node size, greater node quantity, and deeper color signify more robust connections between the node protein and its adjacent proteins. Figure 7B illustrates that the outside circle denotes the targets of active components of highland barley polyphenols, whereas the inner circle signifies the possible targets for hypertension. The network comprises 64 nodes (20 active component nodes and 44 target nodes) and 149 edges, with each edge representing the interaction between an active component of highland barley polyphenols and its respective functional target. Figure 7B suggests that Apigenin 7-O-(2G-rhamnosyl) gentiobioside, Vanillic acid, (−)-Gallocatechin, and other active constituents may be the principal functional components of highland barley polyphenols responsible for antihypertensive effects.

#### 3.4.2. PPI Network and Key Target Identification

The PPI network was established to identify the primary treatment targets for hypertension, based on the 44 antihypertensive targets from 20 polyphenolic compounds of EX-treated highland barley samples, using STRING and Cytoscape (Figure 7C). The PPI network comprised 38 nodes and 148 edges, with each node representing a protein and each edge denoting the interaction between proteins. The size and color of the nodes signify the degree value: larger nodes correspond with higher degree values, while the color gradient from dark to light reflects a reduction in the degree value. The varied nodes and edges indicate that the principal antihypertensive compounds in highland barley may influence numerous targets and pathways. The Centiscape plugin was also used for topological analysis. The medians of Betweenness Centrality, Closeness Centrality, and Edge Count (Degree) for the nodes were 0.009665916, 0.6, and 7, respectively. In total, 14 targets achieved higher medians of the aforementioned three parameters (Table 4). Genes with elevated Degree values had substantial roles within the network, demonstrated more pronounced associations with disease components, and are more probable targets for pharmacological intervention [38]. Genes with increased Degree values as tumor necrosis factor (TNF), hypoxia-inducible factor 1 alpha (HIF1A), interleukin-6 (IL6), angiotensin-converting enzyme (ACE), and matrix metalloproteinase 9 (MMP9), which showed Degree values of 24, 18, 21, 19, and 17, respectively.

Among these targets, TNF is predominantly released by macrophages. It can stimulate apoptosis in some tumor cell lines and is also associated with the onset of cachexia [39]. Verma et al. [40] showed that TNF-α, as an inflammatory cytokine, is essential in the pathophysiology of hypertension. Hypertension promotes multiple organ complications among patients, including left ventricular hypertrophy, heart failure, and renal impairment, and contributes to atherosclerosis development. Lipid management and controlling atherosclerosis may represent the most vital approaches for treating hypertension [41]. IL-6 has been associated with several cardiovascular diseases, such as atherosclerosis, myocarditis, and hypertension. IL-6 may influence the onset of hypertension through mechanisms including modification of leukocyte rheological properties, the increase in intracellular Ca^2+^ levels in vascular smooth muscle cells, stimulation of vascular smooth muscle cell proliferation, and the improvement of peripheral vascular resistance [42]. MMP9 is a protease that degrades the extracellular matrix and substantially contributes to the formation and rupture of atherosclerotic plaques. Research indicates that the MMP9 silencing reduces pulmonary artery pressure and right ventricular systolic pressure, mitigates right ventricular hypertrophy, and thus has an anti-pulmonary arterial hypertension effect [43]. HIF1A is a hypoxia-inducible factor that modulates various genes associated with adaptation to low-oxygen conditions, ultimately enhancing cellular resilience to hypoxia. Tuo et al. [44] identified MMP9 and HIF1A, among others, as significant therapeutic targets for hypertension, validating the findings of this study. ACE facilitates the transformation of angiotensin I (AngI) into angiotensin II (AngII), increasing blood pressure. Furthermore, ACE degrades bradykinin (BK), thus inhibiting its ability to reduce blood pressure [45]. Thus, ACE is crucial for the regulation of blood pressure.

Highland barley polyphenols may regulate the expression of TNF, HIF1A, IL-6, and ACE to facilitate vasodilation, mitigate inflammatory reactions, and decrease MMP9 expression to enhance vascular elasticity and remodeling. Moreover, by modulating pertinent signaling pathways, they can protect endothelial function and suppress excessive sympathetic activity, thus producing antihypertensive effects.

#### 3.4.3. Enrichment Analyses

**Figure 8:**
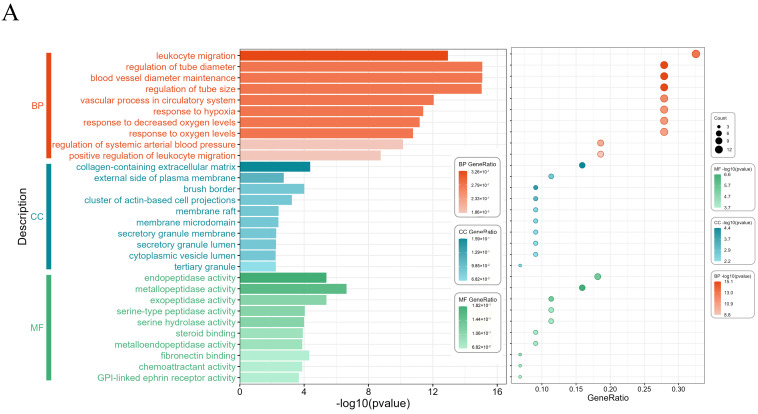

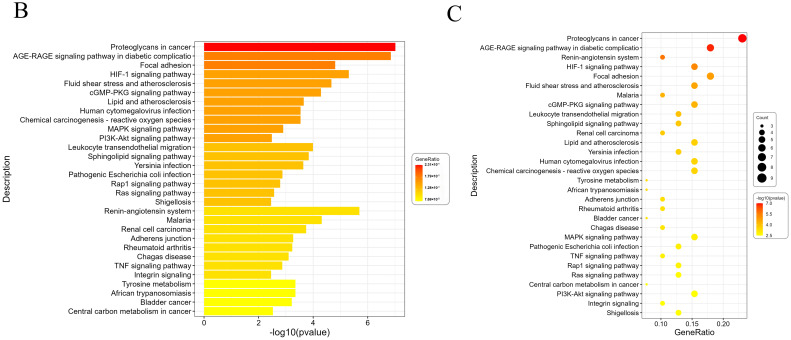
GO and KEGG enrichment analyses of differentially expressed genes. (**A**): GO enrichment analysis, including a bar chart of the top enriched GO terms (grouped into BP, CC, and MF categories) and a corresponding bubble plot; (**B**): bar chart of the top enriched KEGG pathways; (**C**): bubble plot visualization of KEGG pathway enrichment results).

GO functional enrichment revealed 1419 GO terms (*p* < 0.05), comprising 1273 BP, 32 CC, and 114 MF terms. The GO terms for genes were classified according to their *p*-values, and the top ten terms from each category were selected for compiling bar charts (Figure 8A). The y-axis in these plots denotes the quantity of enriched genes. The biological mechanisms largely encompass leukocyte migration, tube diameter regulation, and blood vessel diameter maintenance, which are fundamental pathways implicated in hypertension pathology and its pharmaceutical management. This indicates that highland barley polyphenols may enhance arterial dilatation by modulating vascular tone and inflammatory cell infiltration. Key cellular component words mostly refer to features including the collagen-rich extracellular matrix, the external side of the plasma membrane, and the brush border. These structures are related to the secretion and transport of hormones and neurotransmitters, suggesting that highland barley polyphenols may affect the release of vasoactive molecules, including endothelin and nitric oxide. This indicates that highland barley polyphenols may enhance the elasticity and function of the vascular wall via modulating extracellular matrix remodeling and membrane surface signal transmission. The enriched MF terms included endopeptidase, metallocarboxypeptidase, and serine-type peptidase activities. These enzymes participate in the breakdown of vasoactive peptides, indicating that highland barley polyphenols may regulate the equilibrium between vascular dilatation and constriction by altering enzyme activity.

The “clusterProfiler” package in R was utilized to import core targets for KEGG pathway enrichment analysis, which identified 73 pathways (*p* < 0.05). Supplementary Table S4 presents the top 30 pathways, ranked by *p*-value, along with the genes associated with each pathway and their respective *p*-values. The 30 most significantly enriched pathways were identified based on *p*-values and illustrated in bar charts (Figure 8B) and bubble charts (Figure 8C). Figure 8C predominantly illustrates the tabular result, wherein the size of the circles indicates the ratio of selected genes to the total gene count within the pathway, and the color intensity (towards red) reflects lower original *p*-values, signifying more robust and significant enrichment results. GO and KEGG enrichment analyses indicated that the antihypertensive mechanisms of highland barley polyphenols predominantly target pathways comprising lipid metabolism and atherosclerosis, fluid shear stress and atherosclerosis, the AGE-RAGE axis in diabetic complications, the TNF axis, the HIF-1 axis, and the MAPK axis.

The results from GO and KEGG analyses indicate that highland barley polyphenols may have antihypertensive therapeutic benefits by influencing multiple critical pathways, including the AGE-RAGE, HIF-1, MAPK, and cGMP-PKG axes. The critical functions of these pathways in hypertension management have been thoroughly documented in previous investigations [46,47,48]. The binding of AGE to RAGE activates downstream signaling pathways, including MAPK, which promotes the production of inflammatory cytokines, aggravating vascular endothelial inflammation and damage. The HIF-1 axis is an essential regulator of hypoxia that facilitates angiogenesis, enhances hypoxic resistance, and improves metabolism. Wang et al. [49] emphasized the importance of the HIF-1 axis in the mechanism of Danshen Yin Granules for treating hypoxic pulmonary hypertension, underscoring its significance in pulmonary arterial hypertension. The MAPK axis predominantly governs the proliferation, apoptosis, autophagy, and oxidative stress responses of smooth muscle cells [50]. This pathway has been recognized as an essential factor in cardiovascular disorders, including hypertension and heart failure [51]. The cGMP/PKG axis regulates blood circulation, increases vascular channel activity, and facilitates relaxation of vascular smooth muscle, resulting in arterial dilation [52]. The active constituents of highland barley polyphenols ameliorate hypertension by engaging various pathways in a coordinated fashion. Furthermore, the utilization of highland barley is economically advantageous and lacks adverse effects, rendering it a viable therapeutic alternative for hypertension management.

#### 3.4.4. Construction of the Core Target–Pathway Network

**Figure 9:**
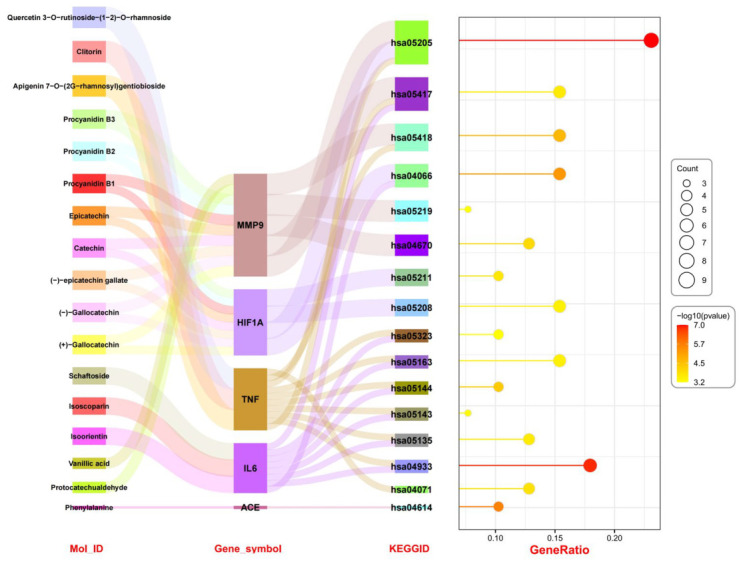
Sankey bubble chart of components–targets–pathways.

The basic target–pathway network was designed utilizing Cytoscape (Supplementary Figure S3). In this network, nodes of larger size and redder color signify a higher level of connection within the interaction network. The network consists of 28 nodes and 40 edges. Figure 9 depicts the relationships between highland barley polyphenolic compounds (Mol ID), principal targets (Gene symbol), and associated KEGG pathways (KEGG ID). The image showed that several polyphenolic compounds concurrently influence core genes such as IL6, TNF, and HIF1A, and are associated with critical pathways, including the AGE-RAGE and the TNF axes. This suggests that the antihypertensive effects of highland barley are mediated by the synergistic interaction of many components, targets, and pathways, aligning with the findings of the enrichment analysis detailed in Section 3.4.3.

### 3.5. Results of Molecular Docking

Molecular docking simulations are used to analyze interactions between receptors and ligands [53]. To investigate the processes responsible for the therapeutic advantages of key phenolic compounds from highland barley on hypertension, Autodock was utilized to determine the binding energies between these compounds and their target proteins. The findings are displayed in Table 5. In general, when the binding energy is <0 kcal/mol, the ligand and receptor can bind spontaneously [54]. A binding energy of <−5 kcal/mol implies possible binding, but a binding energy of <−7.0 kcal/mol indicates a substantial binding affinity [38]. Reduced binding energies indicate an enhanced affinity between the receptor and ligand, more stable conformations of the ligand-receptor complex, and more potential for interaction.

**Figure 10:**
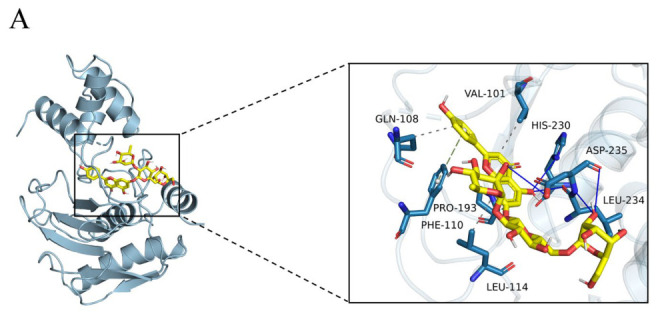

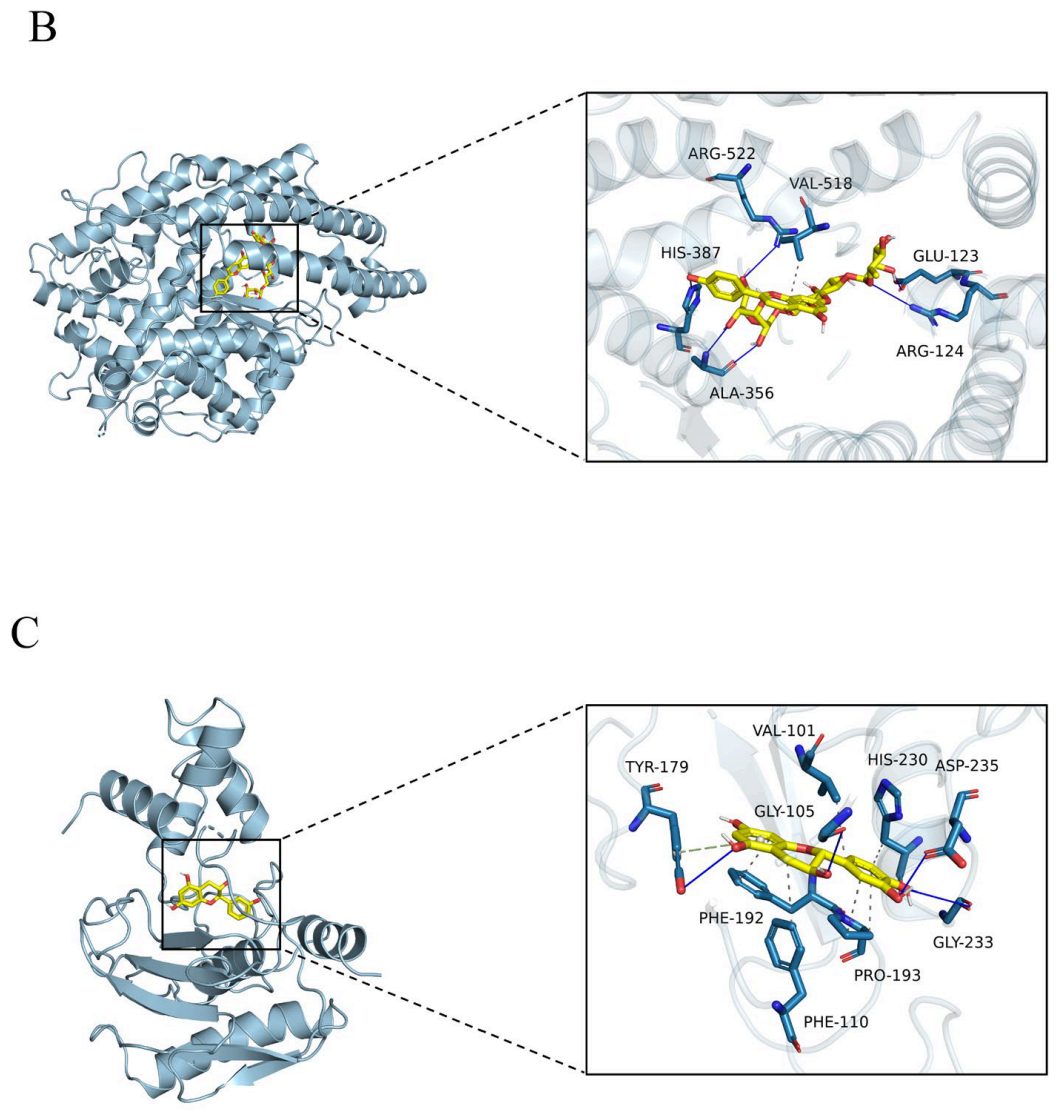
Visualization of molecular docking results.(**A**): Apigenin 7-O-(2G-rhamnosyl)gentiobioside and MMP9; (**B**): Apigenin 7-O-(2G-rhamnosyl) gentiobioside and ACE; (**C**): Catechin and MMP9).

Table 5 indicates that the three highest binding energies are as follows: Apigenin 7-O-(2G-rhamnosyl) gentiobioside demonstrated a binding energy of −9.8 and −9.2 kcal/mol for MMP9 and ACE, respectively, while Catechin binding with MMP9 releases the energy of −8.9 kcal/mol. The top three complexes exhibiting the highest binding energies were selected for visualization to acquire a more intuitive representation of the molecular docking results. Figure 10 displays the visualization results. MMP9 engages with Apigenin 7-O-(2G-rhamnosyl) gentiobioside via hydrophobic interactions involving the protein residues VAL-101, GLN-108, LEU-114, and PRO-193, hydrogen bonds with HIS-230, LEU-234, and ASP-235, and π-π stacking with PHE-110 (Figure 10A). In addition, ACE develops hydrophobic interactions with Apigenin 7-O-(2 G-rhamnosyl) gentiobioside through VAL-518 and generates hydrogen bonds with GLU-123, ARG-124, ALA-356, HIS-387, and ARG-522 (Figure 10B). Moreover, MMP9 engages with Catechin via hydrophobic interactions involving VAL-101, PHE-110, PHE-192, PRO-193, and HIS-230, hydrogen bonds with GLY-105, TYR-179, GLY-233, and ASP-235, and π-π stacking with TYR-179 (Figure 10C). The results indicate that phenolic compounds can efficiently interact with proteins through many mechanisms, such as hydrogen bonding, hydrophobic interactions, and π-π stacking. This binding can inhibit ACE activity to suppress the RAS pathway [55], decrease MMP9 activity to mitigate vascular matrix degradation and remodeling, and regulate the HIF-1/TNF pathways to enhance hypoxic and inflammatory microenvironments. Through these synergistic effects across different pathways, phenolic compounds can facilitate vasodilation, maintain cardiovascular health, and reduce blood pressure.

## 4. Limitations and Recommendations

This study initially developed a framework elucidating the mechanism via which highland barley polyphenols ameliorate hypertension, utilizing network pharmacology. The analytical results were primarily obtained from theoretical data in existing databases and were not experimentally verified using in vitro studies or animal models. Therefore, the conclusions are predictive inferences, necessitating future experimental investigation to evaluate the interactions among essential components and targets, as well as their actual impacts in physiological contexts. Despite certain limitations, this study offers significant theoretical insights and research prospects for additional comprehensive investigation of the mechanisms and experimental validation. Moreover, it indicates that EX treatment is a comparatively optimal way for processing highland barley-based foods. This research gives references for the systematic creation and application of highland barley-based diets and presents novel concepts for food-derived therapies in hypertension.

## 5. Conclusions

In summary, this research integrated UHPLC-Q Exactive HFX/MS technology and network pharmacology methods to investigate the variations in polyphenolic differential metabolites of highland barley subjected to different thermal treatments. Furthermore, network pharmacology was conducted to determine the mechanism by which highland barley polyphenols treat hypertension. It was observed that different thermal treatments had varying impacts on the polyphenol and flavonoid contents of highland barley. Among these, the flavonoid level in PO-treated highland barley was highest (2.93 ± 0.09 mg/g). The EX and SR treatments enhanced polyphenol content (2.88 ± 0.11 and 2.94 ± 0.06 mg/g, respectively). Subsequently, in vitro antioxidant assays were performed, which showed that the DPPH and FRAP values reached their peak after EX treatment, measuring 9.75 ± 0.24 and 2.92 ± 0.02 μmol Trolox/g DW, respectively. The ABTS and hydroxyl radical scavenging rates attained their maxima in the CK group, measuring 13.32 ± 0.25 μmol Trolox/g DW and 93.32% ± 0.28%, respectively. Targeted metabolomics identified 252 phenolic metabolites, comprising 233 flavonoid metabolites and 19 phenolic acid metabolites. The study additionally predicted specific targets and pathways for hypertension alleviation using highland barley polyphenols. Initially, 44 possible action targets were evaluated, which revealed five key targets: TNF, IL6, MMP9, HIF1A, and ACE. Furthermore, GO and KEGG enrichment analysis demonstrated that these genes were markedly concentrated in pathways associated with lipid metabolism and atherosclerosis, fluid shear stress, and the TNF axis, among other relevant pathways. Molecular docking demonstrated that the principal polyphenolic drugs for hypertension treatment interacted with the primary targets via hydrogen bonds and hydrophobic interactions. Apigenin 7-O-(2-rhamnosyl) gentiobioside demonstrated the highest binding energy with MMP9. Meanwhile, EX is an optimal processing method for highland barley food. The findings lay a foundation for the development of highland barley products and present new ideas for dietary intervention in hypertension.

## Figures and Tables

**Table 1:** Gradient elution conditions of mobile phase for phenolic acid analysis.

Time (min)	Mobile Phase A, Ultrapure Water Containing 0.1% (*v*/*v*) Acetic Acid	Mobile Phase B, Acetonitrile Containing 0.1% (*v*/*v*) Acetic Acid
0	90%	10%
2	90%	10%
6	40%	60%
8	40%	60%
8.1	90%	10%
12	90%	10%

**Table 2:** Gradient elution conditions of mobile phase for flavonoid analysis.

Time (min)	Mobile Phase A, Ultrapure Water Containing 0.1% (*v*/*v*) Formic Acid	Mobile Phase B, Methanol Containing 0.1% (*v*/*v*) Formic Acid
0	95%	5%
1	70%	30%
10	20%	80%
14	5%	95%
16	5%	95%
16.1	95%	5%
18	95%	5%

**Table 3:** Metabolites identified in highland barley samples (VIP > 1 and *p* < 0.05).

No.	Metabolite	Chemical Formula	CAS	*p*-Value	VIP
	Phenolic acids (9)				
1	Protocatechualdehyde	C_7_H_6_O_3_	139-85-5	0.011515267466513	1.148
2	4-Hydroxybenzoic acid	C_7_H_6_O_3_	99-96-7	0.000013576493104	1.02754
3	Vanillic acid	C_8_H_8_O_4_	121-34-6	0.021053975339382	2.10677
4	Caffeic acid	C_9_H_8_O_4_	331-39-5	0.000000000055510	2.0507
5	Vanillin	C_8_H_8_O_3_	121-33-5	0.029932956610940	1.81196
6	p-Hydroxycinnamic acid	C_9_H_8_O_3_	7400-08-0	0.000000004321714	1.76939
7	Trans-Ferulic acid	C_10_H_10_O_4_	537-98-4	0.000000012142298	5.35532
8	Sinapic acid	C_11_H_12_O_5_	530-59-6	0.002749409947376	2.03709
9	Benzoic acid	C_7_H_6_O_2_	65-85-0	0.000000085224947	1.0299
	Flavonoids (11)				
10	Schaftoside	C_26_H_28_O_14_	51938-32-0	0.000000001106474	1.14771
11	Isoorientin	C_21_H_20_O_11_	4261-42-1	0.000000002064219	1.28084
12	Apigenin-7-O-(2G-rhamnosyl)gentiobioside	C_33_H_40_O_19_	174284-20-9	0.000000000000001	2.03469
13	Isoscoparin	C_22_H_22_O_11_	20013-23-4	0.000000001041107	1.40109
14	Procyanidin B2	C_30_H_26_O_12_	29106-49-8	0.000000000293123	4.32568
15	Procyanidin B1	C_30_H_26_O_12_	20315-25-7	0.000000000035915	2.74663
16	Procyanidin B3	C_30_H_26_O_12_	23567-23-9	0.014465316838702	4.33396
17	Rutin	C_27_H_30_O_16_	153-18-4	0.000000530409829	1.68416
18	Clitorin	C_33_H_40_O_19_	55804-74-5	0.000000000000000	2.72545
19	Rutin hydrate	C_27_H_30_O_16_·xH_2_O	207671-50-9	0.000000543022372	1.68129
20	Quercetin 3-O-rutinoside-(1→2)-O-rhamnoside	C_33_H_40_O_20_	55696-57-6	0.000000000000001	5.54972
Flavanols (6)					
21	(−)-Epicatechin gallate	C_22_H_18_O_10_	1257-08-5	0.000023855815199	2.783
22	(−)-Epicatechin	C_15_H_14_O_6_	490-46-0	0.000000000000001	2.6383
23	(+)-Gallocatechin	C_15_H_14_O_7_	970-73-0	0.000000000090168	1.63634
24	(±)-Catechin	C_15_H_14_O_6_	7295-85-4	0.000000000000002	5.08773
25	Catechin	C_15_H_14_O_6_	154-23-4	0.000000000337854	1.59534
26	(−)-Gallocatechin	C_15_H_14_O_7_	3371-27-5	0.000000000066324	1.63067
Amino acid (1)					
27	Phenylalanine	C_9_H_11_NO_2_	63-91-2	0.017151621646513	1.16404

**Table 4:** Topological characteristics of key therapeutic targets for hypertension.

No.	Name	Betweenness Centrality	Closeness Centrality	Degree
1	TNF	1.148673674	0.88	24
2	IL6	1.101188689	0.818181818	21
3	ACE	0.8751001	1	19
4	HIF1A	0.195345345	0.717948718	18
5	MMP9	0.48795045	0.75	17
6	SERPINE1	0.045795796	1	14
7	SELE	0.071433934	0.692307692	12
8	F3	0.110235235	0.666666667	11
9	RHOA	0.045045045	0.631578947	11
10	FLT1	0.013325826	1	10
11	MET	0.047672673	1	9
12	ACE2	0.033283283	0.575757576	9
13	AKR1B1	0.082832833	1	8
14	XDH	0.028528529	0.619047619	8

**Table 5:** Binding energies between core antihypertensive components and key targets in highland barley polyphenols.

Compounds	Binding Energy (kcal mol^−1^)				
	TNF	IL6	MMP9	HIF1A	ACE
Apigenin 7-O-(2G-rhamnosyl) gentiobioside	−8.2	−6.6	−9.8	−2.1	−9.2
Vanillic acid	−5.5	−5.8	−7.0	−6.1	−6.1
(−)-Gallocatechin	−7.1	−6.3	−7.9	−7.2	−8.2
Catechin	−8.1	−6.3	−8.9	−7.2	−7.9
Epicatechin	−8.2	−6.1	−8.1	−7.4	−8.0

## Data Availability

The original contributions presented in the study are included in the article/Supplementary Material; further inquiries can be directed to the corresponding author.

## Supplementary Materials

The following supporting information can be downloaded at https://www.mdpi.com/article/10.3390/foods15122095/s1. Figure S1: Pie chart of phenolic compound contents under different heat treatments; Figure S2: Venn diagram of hypertension-highland barley polyphenols; Figure S3: Network diagram of core targets-pathways; Table S1: Antioxidant capacity of highland barley under different treatments; Table S2: 252 Metabolites; Table S3: Significantly differential metabolites obtained by EX-treated; Table S4: Information on the top 30 KEGG pathways; File S1: Detailed information of metabolite standards.
