# Supplementary material for: Elucidation of the Effects of Heat Treatment on Polyphenolic Compounds in Highland Barley and Their Potential Mechanisms of Action in Improving Hypertension Using Targeted Metabolomics, Network Pharmacology, and Molecular Docking
Source: Foods. 2026 Jun 10;15(12):2095. doi: 10.3390/foods15122095 (PMC13297854; doi:10.3390/foods15122095)

Supplementary Figure

Supplementary Figure S1. Pie chart of phenolic compound contents under different heat treatments
Supplementary Figure S2. Venn diagram of hypertension-highland barley polyphenols
Supplementary Figure S3. Network diagram of core targets-pathways

Supplementary Figure S1

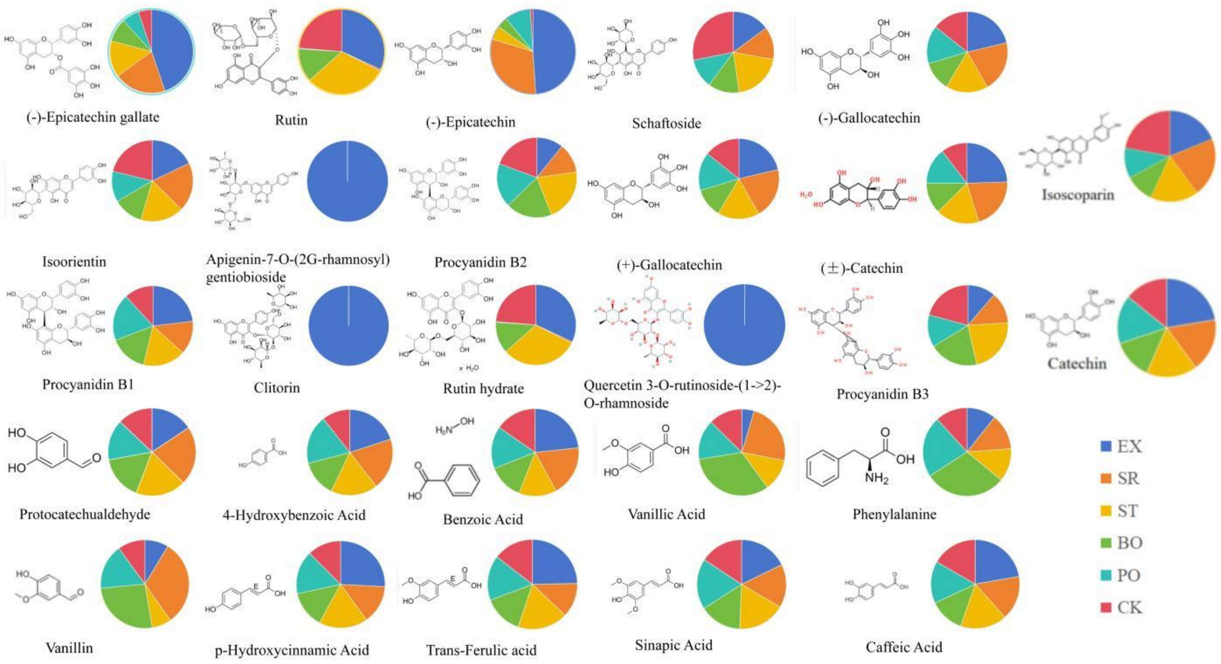

Note: EX: extrusion puffing, SR: sand-roasting, ST: steaming, BO:atmospheric-pressure boiling, PO: high-pressure boiling, CK: Raw

Supplementary Figure S2

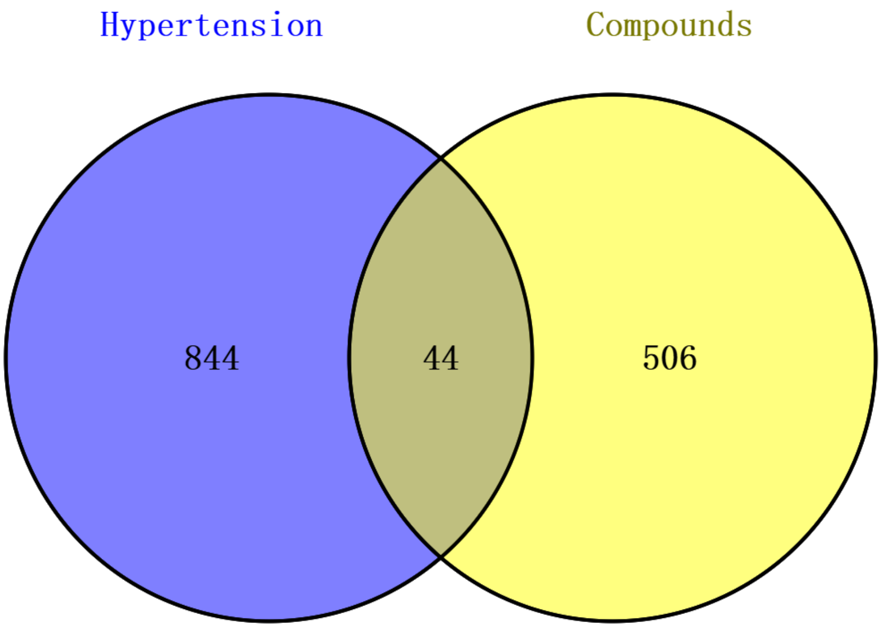

Supplementary Figure S3

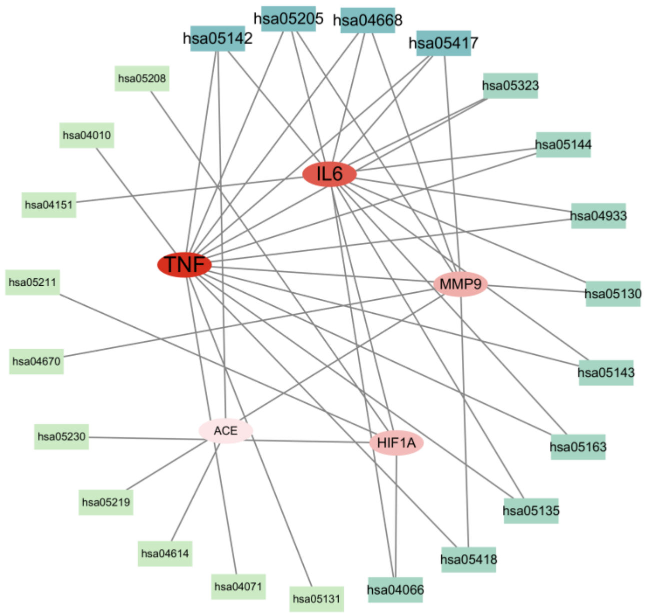

Supplement: Supplementary file 1 [file foods-15-02095-s001.zip › Supplementary Figure.pdf]
