# Supplementary material for: Elucidation of the Effects of Heat Treatment on Polyphenolic Compounds in Highland Barley and Their Potential Mechanisms of Action in Improving Hypertension Using Targeted Metabolomics, Network Pharmacology, and Molecular Docking
Source: Foods. 2026 Jun 10;15(12):2095. doi: 10.3390/foods15122095 (PMC13297854; doi:10.3390/foods15122095)
Supplement: Supplementary file 1 [file foods-15-02095-s001.zip › Supplementary Table.pdf]

Supplementary Table

Supplementary Table S1. Antioxidant capacity of highland barley under different treatments
Supplementary Table S2. 252 Metabolites
Supplementary Table S3. Significantly differential metabolites obtained by EX-treated
Supplementary Table S4. Information on the top 30 KEGG pathways

Supplementary Table S1

Different processing methods	Total Phenols (mg/g DW)	Flavonoids (mg/g DW)	FRAP (μmol Trolox/g DW)	DPPH (μmol Trolox/g DW)	ABTS (μmol Trolox/g DW)	·OH(%)
EX	2.88 ± 0.11^{a}	2.46 ± 0.08^{b}	2.92 ± 0.02^{a}	9.75 ± 0.24^{a}	12.27 ± 0.31^{b}	84.45 ± 0.72^{d}
SR	2.94 ± 0.06^{a}	2.44 ± 0.05^{b}	2.45 ± 0.06^{b}	9.16 ± 0.18^{b}	10.15 ± 0.27^{c}	89.75 ± 0.19^{b}
ST	2.45 ± 0.12^{c}	2.48 ± 0.06^{b}	2.28 ± 0.04^{c}	7.36 ± 0.08^{e}	12.33 ± 0.47^{b}	87.81 ± 0.26^{c}
BO	2.1 ± 0.01^{d}	2.33 ± 0.04^{c}	1.83 ± 0.05^{d}	7.59 ± 0.16^{de}	9.83 ± 0.25^{c}	88.44 ± 0.14^{c}
PO	2.35 ± 0.08^{c}	2.93 ± 0.09^{a}	2.28 ± 0.01^{c}	7.87 ± 0.17^{d}	8.71 ± 0.16^{d}	84.63 ± 0.28^{d}
CK	2.61 ± 0.07^{b}	1.89 ± 0.02^{d}	2.3 ± 0.07^{c}	8.59 ± 0.14^{c}	13.32 ± 0.25^{a}	93.32 ± 0.26^{a}

Supplementary Table S2. 252 Metabolites

No.	Metabolite	No.	Metabolite
1	Scutellarin	55	7-Hydroxyflavone
2	Prunin	56	1,3,5-Trimethoxybenzene
3	Ginkgetin	57	Reynoutrin
4	Hyperoside	58	6-Demethoxytangeretin
5	Daidzin	59	3'-Methoxyflavonol
6	(-)-Epicatechin gallate	60	2-Hydroxychalcone
7	Amentoflavone	61	Visnagin
8	Cynaroside	62	EGCG Octaacetate
9	Xanthone	63	Quercetagetin
10	Baicalein	64	Kaempferol-3-O-glucorhamnoside
11	Naringenin	65	3,6-Dihydroxyflavone
12	Chrysin	66	2-Hydroxyflavanone
13	Tricin	67	4-methoxychalcone
14	Scutellarein	68	Isoliquiritin apioside
15	Genkwanin	69	Glabrol
16	Oroxylin A	70	Rhodionin
17	Apigenin 7-glucoside	71	Kumatakenin
18	(2S)-Isoxanthohumol	72	Hispidulin
19	5,7-Dihydroxychromone	73	Sakuranetin
20	Mosloflavone	74	Miquelianin
21	Norwogonin	75	(±)-Catechin
22	3-Methoxyflavone	76	5,7,4'-Trimethoxyflavone
23	7-Hydroxyisoflavone	77	Nepetin
24	Dihydromyricetin	78	Skullcapflavone II
25	Flavanone	79	3,5,6,7,8,3',4'-Heptamethoxyflavone
26	Daidzein	80	Desmethylglycitein
27	Myricitrin	81	Karanjin
28	Biochanin A	82	Hamaudol
29	Flavonol	83	Procyanidin B1
30	Flavone	84	Sulfuretin
31	6-Hydroxyflavone	85	Mulberrin
32	4-Hydroxychalcone	86	Vincetoxicoside B
33	Hesperetin	87	5,7,3'-Trihydroxy-6,4',5'-trimethoxyflavone
34	Pinocembrin	88	Noreugenin
35	Fisetin	89	3,4-Dihydroxyflavone
36	Puerarin	90	Rhamnocitrin
37	Rutin	91	Luteolin-3-O-beta-D-glucuronide
38	Quercetin	92	Dihydrodaidzein
39	17α-Hydroxyprogesterone	93	Quercimeritrin
40	Khellin	94	Taxifolin 7-O-rhamnoside
41	Diosmetin	95	Luteolinidin chloride
42	Genistein	96	Grosvenorine
43	Bavachinin	97	Lonicerin
44	Wogonin	98	6-Methoxyflavone
45	Isorhamnetin	99	Gardenin B
46	(-)-Epicatechin	100	7,3',4'-Tri-O-methylluteolin
47	Genistin	101	Kaempferol 3-neohesperidoside
48	Nobiletin	102	Chrysoeriol
49	7,8-Dihydroxyflavone	103	Tamarixetin
50	Rhoifolin	104	Swertisin
51	Quercitrin	105	Procyanidin A1
52	Tangeretin	106	(-)-Butin
53	Medroxyprogesterone Acetate	107	Procyanidin A2
54	Sophoricoside	108	Maohuoside A

No.	Metabolite	No.	Metabolite
109	4',7-Dimethoxyisoflavone	161	Clitorin
110	Liquiritigenin	162	Neochamaejasmine B
111	5-methoxyflavone	163	3'-Demethylnobiletin
112	Cimifugin	164	Rutin hydrate
113	Glabridin	165	Tetramethylkaempferol
114	Baohuoside I	166	Poriol
115	trans-Chalcone	167	Pinostrobin
116	Galangin	168	Keracyanin chloride
117	Quercetin Dihydrate	169	Quercetin 3-O-rutinoside-(1-2)-O-rhamnoside
118	Bavachin	170	Lupiwighteone
119	Typhaneoside	171	Kaempferol 3-O-β-D-galactopyranoside
120	Epimedin A	172	Ombuin
121	Oroxin B	173	Prudomestin
122	Tectorigenin	174	(2S)-6-Prenylnaringenin
123	Schaftoside	175	Pratensein
124	Isorhamnetin-3-O-neohespeidoside	176	4',5-Dihydroxyflavone
125	Jaceosidin	177	5-hydroxy-3,7-dimethoxy-2-phenylchr omen-4-one
126	Isobavachin	178	S-Dihydrodaidzein
127	(-)-Gallocatechin	179	3',4',7-Trimethoxyquercetin
128	Irigenin	180	Robinetin
129	Dracorhodin perchlorate	181	Leachianone A
130	Didymin	182	Ternatumoside II
131	Nicotiflorin	183	3',4',7-Trihydroxyflavone
132	Homoplantaginin	184	Hydrangenol
133	Maackiain	185	Chrysosplenol D
134	Diosmetin-7-O-β-D-glucopyranoside	186	Ayanin
135	Hydroxygenkwanin	187	Rhamnetin
136	Tiliroside	188	5,7-Dimethoxyluteolin
137	Apiin	189	Kuwanon H
138	Orientin	190	3-O-Methylquercetin
139	Kurarinone	191	Afzelin
140	Vitexin	192	Meloside A
141	β-Anhydroicaritin	193	Aureusidin
142	Glycitein	194	Scutellarein tetramethyl ether
143	Irisflorentin	195	Cirsimaritin
144	Isoorientin	196	Kaempferol-7,4'-dimethyl ether
145	Astragalin	197	5,7-Diacetoxy-8-methoxyflavone
146	Linarin	198	Isoscoparin
147	Isosakuranetin	199	6-Hydroxyluteolin 7-glucoside
148	Isoquercetin	200	4-methyl-6-phenyl-2H-pyranone
149	Isorhamnetin-3-O-glucoside	201	Chamaechromone
150	Pectolinarigenin	202	Luteolin
151	Tectochrysin	203	Apigenin
152	Prunetin	204	Isovitexin
153	Narcissin	205	4'-Hydroxychalcone
154	Morusin	206	3'-Methoxyapiin
155	(±)-Naringenin	207	Procyanidin B3
156	Methylophiopogonanone B	208	Vitexin-4''-O-glucoside
157	Sanggenon C	209	4',6,7-Trimethoxyisoflavone
158	5-Methyl-7-methoxyisoflavone	210	Taxifolin
159	Epimedin A1	211	Naringin
160	Apigenin-7-O-(2G-rhamnosyl)gentiobi oside	212	Deguelin

No.	Metabolite	No.	Metabolite
213	Farrerol	233	Cyanidin Chloride
214	6'''-Feruloylspinosin	234	Gallic acid
215	Sophoraflavanone G	235	Phenylalanine
216	Calycosin	236	3,4-Dihydroxybenzoic acid
217	Luteolin 7-O-glucuronide	237	Protocatechualdehyde
218	Procyanidin B2	238	4-Hydroxybenzoic acid
219	(+)-Gallocatechin	239	Catechin
220	Kaempferol-7-O-β-D-glucopyranoside	240	Vanillic acid
221	Iridin	241	Caffeic acid
222	Brazilin	242	Syringic acid
223	ISOGINKGETIN	243	L-Epicatechin
224	Complanatuside	244	Vanillin
225	Oroxin A	245	p-Hydroxycinnamic Acid
226	Naringenin trimethyl ether	246	Syringaldehyde
227	Corylin	247	Trans-Ferulic acid
228	5-O-Demethylnobiletin	248	Sinapic Acid
229	3-O-Methylgalangin	249	Salicylic acid
230	2''-O-beta-L-galactopyranosylorientin	250	Benzoic acid
231	7,4'-Di-O-methylapigenin	251	Hydrocinnamic acid
232	5,7-dimethoxyflavone	252	Trans-Cinnamic acid

Supplementary Table S3

No.	Name	CAS	Chemical formula
1	Apigenin 7-O-(2G-rhamnosyl)gentiobioside	174284-20-9	$C_{33}H_{40}O_{19}$
2	Vanillic acid	121-34-6	$C_8H_8O_4$
3	(-)-Gallocatechin	3371-27-5	$C_{15}H_{14}O_7$
4	Catechin	154-23-4	$C_{15}H_{14}O_6$
5	Epicatechin	490-46-0	$C_{15}H_{14}$O6
6	(+)-Gallocatechin	970-73-0	$C_{15}H_{14}O_7$
7	(-)-epicatechin gallate	1257-08-5	$C_{22}H_{18}O_{10}$
8	Benzoic acid	65-85-0	$C_7H_6O_2$
9	Clitorin	55804-74-5	$C_{33}H_{40}O_{19}$
10	Quercetin 3-O-rutinoside-(1-2)-O-rhamnoside	55696-57-6	$C_{33}H_{40}O_{20}$
11	Phenylalanine	167088-01-9	$C_9H_{11}NO_2$
12	Procyanidin B1	20315-25-7	$C_{30}H_{26}O_{12}$
13	Isoorientin	4261-42-1	$C_{21}H_{20}O_{11}$
14	Isoscoparin	20013-23-4	$C_{22}H_{22}O_{11}$
15	Procyanidin B2	29106-49-8	$C_{30}H_{26}O_{12}$
16	Procyanidin B3	23567-23-9	$C_{30}H_{26}O_{12}$
17	Schaftoside	51938-32-0	$C_{26}H_{28}O_{14}$
18	Protocatechualdehyde	139-85-5	$C_7H_6O_3$
19	4-Hydroxybenzoic acid	99-96-7	$C_7H_6O_3$
20	Vanillin	121-33-5	$C_8H_8O_3$

Supplementary Table S4

KEGGID	*P* value	Gene ID	Gene_symbol
hsa05205	9.68154636285876e-08	4313/4233/3091/4318/7422/7124/387/6093/9475	MMP2/MET/HIF1A/MMP9/VEGFA/TNF/RHOA/ROCK1/ROCK2
hsa04933	1.42635439334065e-07	4313/7422/7124/5054/3569/6401/2152	MMP2/VEGFA/TNF/SERPINE1/IL6/SELE/F3
hsa04614	2.01186861239697e-06	59272/1636/2028/4311	ACE2/ACE/ENPEP/MME
hsa04066	4.95854193694525e-06	3091/7422/2321/5054/54583/3569	HIF1A/VEGFA/FLT1/SERPINE1/EGLN1/IL6
hsa04510	1.55579715280563e-05	4233/5228/7422/2321/387/6093/9475	MET/PGF/VEGFA/FLT1/RHOA/ROCK1/ROCK2
hsa05418	2.15267522880861e-05	4313/4318/7422/7124/6401/387	MMP2/MMP9/VEGFA/TNF/SELE/RHOA
hsa05144	4.83281537154167e-05	4233/7124/3569/6401	MET/TNF/IL6/SELE
hsa04022	5.19451853922368e-05	134/152/8654/387/6093/9475	ADORA1/ADRA2C/PDE5A/RHOA/ROCK1/ROCK2
hsa04670	0.000101780597076973	4313/4318/387/6093/9475	MMP2/MMP9/RHOA/ROCK1/ROCK2
hsa04071	0.000144889060930977	134/7124/387/6093/9475	ADORA1/TNF/RHOA/ROCK1/ROCK2
hsa05211	0.000181422518586878	4233/3091/7422/54583	MET/HIF1A/VEGFA/EGLN1
hsa05417	0.000221790901007023	4318/7124/3569/6401/387/9475	MMP9/TNF/IL6/SELE/RHOA/ROCK2
hsa05135	0.000230355026569794	7124/3569/387/6093/9475	TNF/IL6/RHOA/ROCK1/ROCK2
hsa05163	0.000290112922693472	7422/7124/3569/387/6093/9475	VEGFA/TNF/IL6/RHOA/ROCK1/ROCK2
hsa05208	0.000290112922693472	4233/3091/7422/2052/2053/4705	MET/HIF1A/VEGFA/EPHX1/EPHX2/NDUFA10
hsa00350	0.000448286383913235	1312/8639/1621	COMT/AOC3/DBH
hsa05143	0.000448286383913235	7124/3569/6401	TNF/IL6/SELE
hsa04520	0.000539979979474337	4233/387/6093/9475	MET/RHOA/ROCK1/ROCK2
hsa05323	0.000585323001810555	7422/2321/7124/3569	VEGFA/FLT1/TNF/IL6
hsa05219	0.000608102016451667	4313/4318/7422	MMP2/MMP9/VEGFA
hsa05142	0.000794028378342596	7124/5054/1636/3569	TNF/SERPINE1/ACE/IL6
hsa04010	0.00126266496388976	4233/5228/7422/2321/7124/5979	MET/PGF/VEGFA/FLT1/TNF/RET
hsa05130	0.0013384630048631	7124/3569/387/6093/9475	TNF/IL6/RHOA/ROCK1/ROCK2
hsa04668	0.00136072270253623	4318/7124/3569/6401	MMP9/TNF/IL6/SELE
hsa04015	0.00162211308786462	4233/5228/7422/2321/387	MET/PGF/VEGFA/FLT1/RHOA
hsa04014	0.00269103159230376	4233/5228/7422/2321/387	MET/PGF/VEGFA/FLT1/RHOA
hsa05230	0.00299557882721917	4233/3091/5979	MET/HIF1A/RET
hsa04151	0.00326490891254814	4233/5228/7422/2321/3569/5979	MET/PGF/VEGFA/FLT1/IL6/RET
hsa04518	0.00348501676591467	4233/5228/7422/2321	MET/PGF/VEGFA/FLT1
hsa05131	0.00350213013579431	7124/826/387/6093/9475	TNF/CAPNS1/RHOA/ROCK1/ROCK2
